# Extremely Low Concentrations of Acetic Acid Stimulate Cell Differentiation in Rice Blast Fungus

**DOI:** 10.1016/j.isci.2019.100786

**Published:** 2019-12-19

**Authors:** Misa Kuroki, Yuriko Shiga, Megumi Narukawa-Nara, Takayuki Arazoe, Takashi Kamakura

**Affiliations:** 1 Tokyo University of Science, Department of Applied Biological Science, Faculty of Science and Technology, 2641, Yamazaki, Noda, Chiba 278-8510, Japan; 2 Osaka University, Research Institute for Microbial Diseases, Department of Molecular Microbiology, 3-1 Yamadaoka, Suita, Osaka 565-0871 Japan

**Keywords:** Biological Sciences, Plant Biology, Interaction of Plants with Organisms, Molecular Plant Pathology, Plant Pathology

## Abstract

Metabolic switching and rewiring play a dynamic role in programmed cell differentiation. Many pathogenic microbes need to survive in nutrient-deficient conditions and use the glyoxylate cycle, an anaplerotic pathway of the tricarboxylic acid cycle, to produce carbohydrates. The plant pathogenic fungus *Magnaporthe oryzae* (*Pyricularia oryzae*) has a unique chitin deacetylase, Cbp1. The spatiotemporal activity of this protein is required for modification of the *M. oryzae* wall and for cell differentiation into the specialized infection structure (appressorium). Here we show that acetic acid, another product released by the Cbp1-catalyzed conversion of chitin into chitosan, induces appressorium formation. An extremely low concentration (fM) of acetic acid restored cell differentiation in a Δ*cbp1* mutant possibly through the glyoxylate cycle.

## Highlights

• Acidification occurred by chitin deacetylase activity during cell differentiation
• Extremely low concentrations of exogenous acetic acid stimulated cell differentiation
• Exogenous acetic acid induced *ICL1* expression, a member of the glyoxylate cycle
• Deletion of *ICL1* inhibited acetic acid-mediated cell differentiation

## Graphical Abstract

**Figure:**
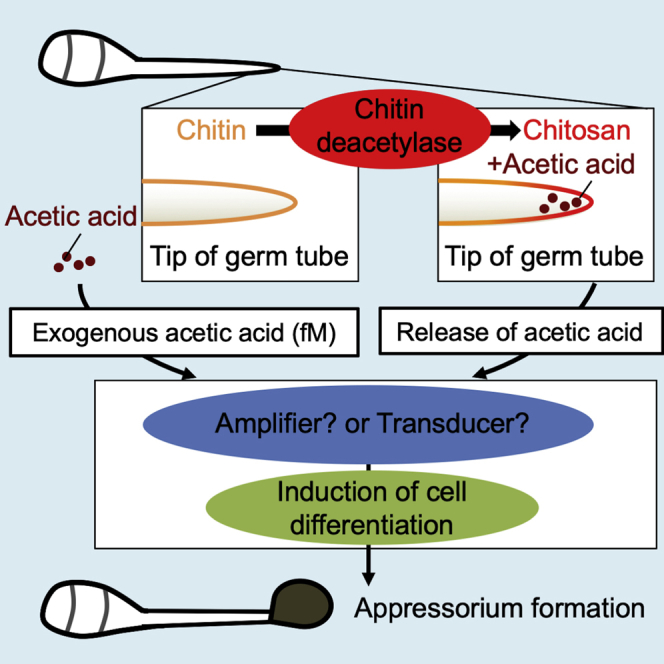

## Introduction

All living organisms and their individual cells must respond and adapt to various environmental changes. In particular, energy substrate starvation conditions dramatically influence intracellular metabolism. Recent studies have uncovered that the nutrient environment, metabolic switching, and metabolites are not only involved in maintaining cellular homeostasis but also play a central role in regulating programmed cell differentiation by acting as donors, substrates, and cofactors for DNA and histone modifications (Carey et al., 2015, Moussaieff et al., 2015, Wellen et al., 2009). Pathogenic microbes are often exposed to nutrient-deficient environments during invasion, and many pathogens use the glyoxylate cycle to assimilate fatty acids for the production of carbohydrates (Lorenz and Fink, 2001, McKinney et al., 2000, Idnurm and Howlett, 2002, Wang et al., 2003, Dunn et al., 2009). The glyoxylate cycle is an anaplerotic pathway of the tricarboxylic acid (TCA) cycle and has a unique two-step metabolic bypass pathway using isocitrate lyase (Icl) and malate synthase (Mls) (Figure S1). In this cycle, acetyl-CoA derived from fatty acids is catalyzed by citrate synthase, which is converted to isocitrate. Icl catalyzes the conversion of isocitrate to glyoxylate and succinate, and glyoxylate is further catalyzed by Mls, which results in malate. Succinate is also used in gluconeogenesis via the TCA cycle. This bypass is reported to be essential for the virulence of many pathogenic microbes (Lorenz and Fink, 2001, McKinney et al., 2000, Idnurm and Howlett, 2002, Wang et al., 2003, Dunn et al., 2009). Metabolic switching in nutrient-deficient environments is dependent on changes in the nutrient source (Nakatsukasa et al., 2015), but its mechanism remains poorly understood.

**Figure 1:**
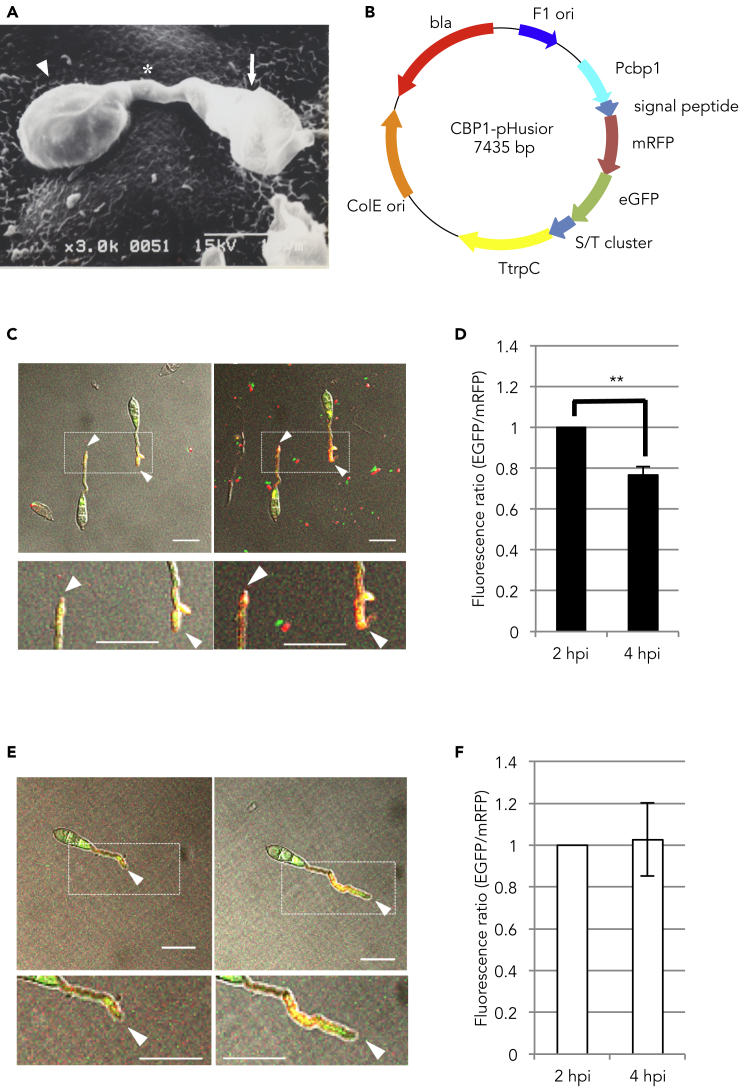
Effect of pH on Appressorium Formation and pH Shift Observations during Germ Tube Elongation (A) Electron microscopic image of appressorium formation in *Magnaoporthe oryzae*. Arrow, asterisk, and arrowhead point to conidium, germ tube, and appressorium, respectively. The conidium elongates the germ tube and forms appressorium at the tip of the germ tube. Scale bar, 10 μm. (B) Schematic representation of the pHusion vector designed for the spatiotemporal expression of the mRFP-eGFP fusion gene. (C) Confocal observations of the pH shift during germ tube elongation in the wild-type strain expressing the pHusion system. Shown are confocal images of the merged differential interference contrast and fluorescence at 2 (left panels) and 4 hpi (right panels). Magnified views of the region are indicated by a white square shown below (lower panels). Arrowheads point to the tip of the germ tube. Scale bar, 20 μm. See also Figures S2 and S3. (D) Fluorescence ratio (eGFP/mRFP) at the tip of the germ tube in the wild-type strain expressing the pHusion system. The observations were repeated 11 times. **p < 0.01 (Student's *t*-test). Error bars show standard deviations. (E) Confocal observations of the pH shift in the Δ*cbp1* mutant expressing the pHusion system. Arrowheads point to the tip of the germ tube. Scale bar, 20 μm. See also Figure S4. (F) Fluorescence ratio (eGFP/mRFP) at the tip of the germ tube in the Δ*cbp1* mutant expressing the pHusion system. Observations were repeated 11 times. Error bars show standard deviations.

The plant pathogenic fungus *Magnaporthe oryzae* (*Pyricularia oryzae*) causes rice blast, the most devastating and economically significant disease of cultivated rice worldwide (Kumar et al., 2013). To infect plant cells, *M. oryzae* develops a specialized dome-shaped structure known as an appressorium that generates an enormous turgor pressure and produces a penetration peg to break the plant cell wall and invade the epidermal cell (Bourett and Howard, 1990, Xu and Hamer, 1996, Zhao and Xu, 2007, Howard et al., 1991, Money and Howard, 1996) (Figure 1A). Appressorium develops at the tip of a polarized germ tube, and this morphogenesis is accompanied by single cell differentiation and cell division. During cell differentiation, the tip of the germ tube recognizes several physical and chemical extracellular cues such as surface hydrophobicity, hardness, host plant-derived chemicals, and other surface conditions (Jelitto et al., 1994, Lee and Dean, 1994, Liu et al., 2007, Xiao et al., 1994, Gilbert et al., 1996). The received cues are transmitted to pivotal signal transduction pathways, namely, the cyclic AMP (cAMP)-dependent kinase and mitogen-activated protein (MAP) kinase signaling cascades, inducing cell differentiation (Li et al., 2012). However, not all cues are required for appressorium formation. For example, appressorium formation can be induced solely by the hydrophobic surface and hardness of the artificial hydrophobic surface in the absence of plant components. Conversely, appressorium formation can be induced by plant wax and cutin without cues associated with surface hydrophobicity and hardness of the plant surface. Therefore, *M. oryzae* is a useful model organism for the study of both plant pathogenic fungi and cell differentiation in multicellular organisms.

Chitin-binding protein, Cbp1, is one of the components involved in signaling pathways associated with appressorium formation in *M. oryzae*. A *CBP1* deletion mutant showed significantly delayed appressorium formation relative to the wild-type strain on hydrophobic surfaces. Cbp1 has a signal peptide and Ser/Thr cluster and localizes to the cell surface (Kamakura et al., 2002). Most recently, Kuroki et al*.* revealed that the chitin deacetylase (CDA) activity of Cbp1 plays a key role in the early phase of cell differentiation in *M. oryzae* (Kuroki et al., 2017). Generally, CDAs convert chitin into chitosan and some fungi harbor *CDA* genes in their genomes. Chitin is a major component of most fungal cell walls, and chitin oligosaccharides decomposed by a plant chitinase are recognized by chitin-specific receptors, triggering further plant defense responses (Shimizu et al., 2010). Thus, in plant pathogenic fungi, it is thought that CDA plays a role in converting their own cell walls via acetylation to prevent plant defense responses. A recent study showed that an exogenous chitosan restored appressorium formation in a Δ*cbp1* mutant, supporting the conclusion that the CDA activity of Cbp1 is required for appressorium development, not for evading the host defense system (Geoghegan and Gurr, 2016). However, both chitin and chitosan consist of *N*-acetyl-D-glucosamine and D-glucosamine, and chitosan can be characterized by the degree of its deacetylation (Brugnerotto et al., 2001). In this study, we focused on acetic acid, which is another product released by the Cbp1-catalyzed conversion of chitin into chitosan, as a candidate for the upstream factor of signal transduction for infection-specific cell differentiation. This study suggests that the extremely low concentration (fM) of acetic acid induces cell differentiation, and that it may influence the glyoxylate cycle. The role of acetic acid at concentrations below the limits of detection has been neglected for a long time. Here, we provide evidence that ultra-trace concentrations of acetic acid stimulate several cellular activities in the rice blast fungus.

## Results

### pH Shift at the Tip of the Germ Tube Mediated by Cbp1 Activity Is Required for Normal Appressorium Formation

We hypothesized that acetic acid, released by CDA activity, plays an important role as a candidate upstream factor of signal transduction for cell differentiation in *M. oryzae*. Acetic acid is a low-molecular-weight short-chain fatty acid that is thought to diffuse immediately after it is generated, thereby affecting the environmental pH (Walter et al., 1982). If acetic acid is released via CDA-catalyzed conversion of chitin into chitosan, acidification is likely to occur at the tip of the germ tube during appressorium differentiation because Cbp1 localizes to the cell surface at the tip of the germ tube. To detect this occurrence in living cells, we used the pHusion system (Gjetting et al., 2012), comprising a tandem fusion gene of eGFP (low pH sensitivity) and mRFP (pH insensitive), as a pH indicator. In this system, the pH shift can be determined from the ratio of eGFP to mRFP fluorescence. To validate whether the spatiotemporal Cbp1 activity induces acidification at the tip of the germ tube, the eGFP-mRFP gene was fused with the signal peptide and S/T cluster derived from Cbp1 and expressed under the endogenous *CBP1* promoter (Figure 1B). This meant that the fusion gene product was transported to the cell surface along with Cbp1. Conidia were collected from the wild-type strain, and eGFP and mRFP fluorescence was observed at 2 and 4 hours post-inoculation (hpi) on an artificial hydrophobic surface, which can induce appressorium formation. These time points corresponded to the previously observed chitosan accumulation via Cbp1 activity (Kuroki et al., 2017). In the wild-type strain expressing the modified pHusion system, a decrease in the fluorescence ratio of eGFP to mRFP was observed at 4 hpi relative to that at 2 hpi (Figures 1C, 1D, and S2). The detected acidification was consistent with the phase of appressorium formation and Cbp1 localization (Figure S3). We also performed the pH shift assay in the Δ*cbp1* mutant expressing the pHusion system, but acidification was not observed at 4 hpi (Figures 1E, 1F, and S4). We did not observe any significant shift in the pH of the harvested conidia or appressoria in the wild-type strain, suggesting that Cbp1 specifically functions at the tip of the germ tube to induce appressorium formation. This result is consistent with the localization and function of Cbp1 (Geoghegan and Gurr, 2016, Kuroki et al., 2017). In addition, because appressorium formation is significantly delayed in the Δ*cbp1* mutant, we could not compare acidification of the appressorium between the wild-type and Δ*cbp1* mutant strains at the same time point. Although the pH shift is not always caused only by acetic acid, and because Cbp1 activity might indirectly affect the levels of ions other than acetic acid, these results suggested that the pH shift at the tip of the germ tube is due to the release of acetic acid linking with CDA activity, and the acetic acid-mediated pH shift can potentially trigger appressorium formation.

### Extremely Low Concentrations of Acetic Acid, Propionic Acid, and Sorbic Acid Induce Appressorium Formation in the Δ*cbp1* Mutant

**Figure 2:**
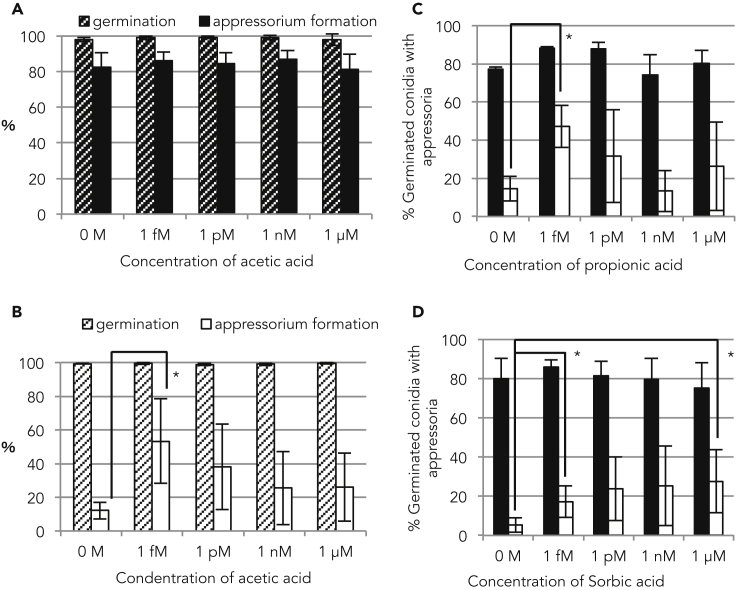
Extremely Low Concentration of Acetic Acid, Propionic Acid, and Sorbic Acid Induce Appressorium Formation Each acid was added at each concentration as shown on the x axis. (A and B) Effects of acetic acid on germ tube elongation and appressorium formation. Diagonal bars indicate the rates of conidia germination, and filled bars indicate the rates of conidia with appressorium formation in the wild-type (A) and Δ*cbp1* mutant (B). Experiments were repeated three times. *p < 0.05 (Student's t test). Error bars show standard deviations. (C and D) Effects of propionic acid (C) and sorbic acid (D) on appressorium formation. Black bars indicate the appressorium formation rate of the wild-type strain, and white bars indicate that of the Δ*cbp1* mutant. *p < 0.05 (Student's t test). Error bars show standard deviations. See also Table S1 and Figures S5–S7.

Next, to test whether acetic acid is directly involved in appressorium differentiation in *M. oryzae*, exogenous acetic acid was added directly to the conidial suspension, and appressorium formation was evaluated. It was found that 1 mM acetic acid severely inhibited both conidial germination and appressorium formation in the wild-type strain (Figure S5). This result was consistent with acetic acid suppressing fungal growth (Kang et al., 2003, Peláeza et al., 2012, Hassan et al., 2015). Thus, we diluted the concentration of acetic acid to 1 μM, 1 nM, 1 pM, and 1 fM. Exposure of conidia to each of these dilutions had no effect on conidial germination or appressorium formation in the wild-type strain (Figure 2A). In contrast, in the Δ*cbp1* mutant, acetic acid could restore appressorium formation; surprisingly, this restoration was observed by adding an extremely low concentration (1 fM) of acetic acid (Figure 2B). This phenotypic restoration indicated that acetic acid partially complemented the loss of Cbp1 activity and that acetic acid released by Cbp1 induced appressorium differentiation in *M. oryzae*. We examined whether each concentration of acetic acid could change the pH of the spore suspension. Although the 1 mM acetic acid solution changed the pH value to 3.77, adding a low concentration of acetic acid (≤1 μM) resulted in a pH value of around 6.0. Based on this result, we predicted that the difference in appressorium formation rate is dependent on the concentration of acetic acid. Thus, it appears that secondary messenger or metabolite activity, rather than involvement in the release of hydrogen ions, is the more important role of low concentrations of acetic acid in promoting appressorium formation in the Δ*cbp1* mutant.

We then investigated whether other acids could restore appressorium formation in *M. oryzae* because it seemed that the pH shift and/or structure of acetic acid played a role as a signaling factor for appressorium formation. Formic acid, propionic acid, butyric acid, oxalic acid, glycine, malonic acid, lactic acid, and succinic acid were selected as carboxylic acids resembling the structure of acetic acid. We also selected sorbic acid and citric acid as mild acids and phosphoric acid and hydrochloric acid as strong acids. The structures of these acids differ from that of acetic acid. The structures of all acids used in this study are depicted in Figure S6. Each acid was also concentrated to 1 μM–1 fM, and the rates of appressorium formation relating to each of these acids were evaluated. In the wild-type strain, with the exclusion of hydrochloric acid, the inhibition of appressorium formation was not observed at acid concentrations lower than 1 μM, and this included acetic acid. In the Δ*cbp1* mutant, low concentrations of propionic acid or sorbic acid could restore appressorium formation (Figures 2C and 2D), but the other acids showed no such phenotypic restoration (Figure S7). Interestingly, propionic acid is structurally similar to acetic acid but sorbic acid shares low similarity to each. When focusing on pKa, all acids that can restore cell differentiation have relatively high pKa values (Table S1). Generally, when the pH is lower than the pKa value, acids exist in a lipophilic undissociated state and show high membrane permeability (Roe et al., 2002). Butyric acid, which has a high pKa value, also showed a tendency to restore appressorium formation of the Δ*cbp1* mutant, although there were no significant differences in the presence or absence of the acids (Figure S7C). These results indicated that not only acetic acid but also the other fatty acids can restore appressorium formation and suggested that this was attributable to the acids being in an undissociated state.

**Figure 3:**
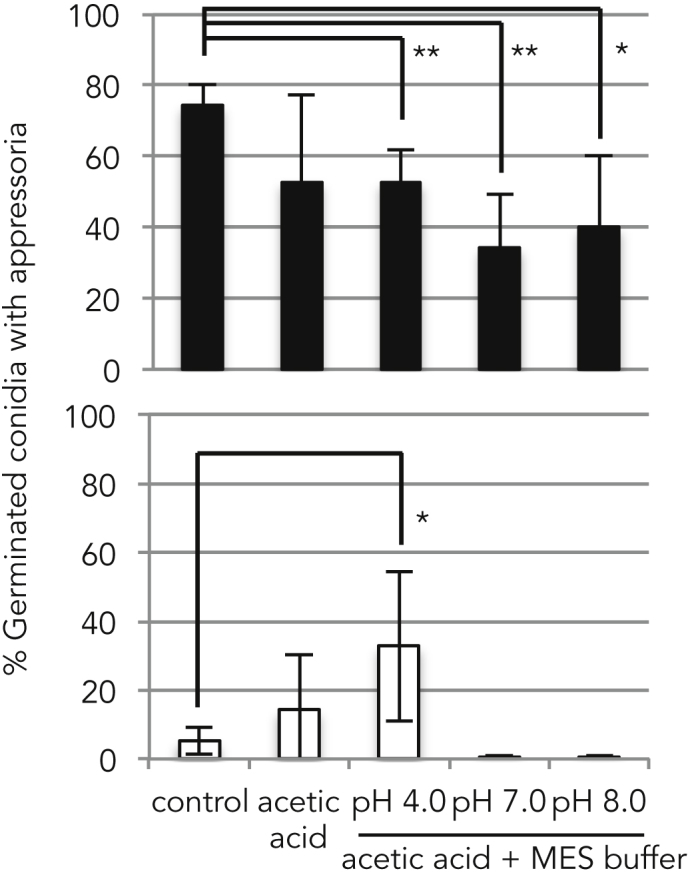
Undissociated State of Acetic Acid Induces Appressorium Formation Black bars indicate the wild-type strain, and white bars indicate the Δ*cbp1* mutant. MES buffer was adjusted to a range of pH values as shown on the x axis. Experiments were repeated five times. *p < 0.05, **p < 0.01 (Student's t test). Error bars indicate standard deviations. See also Figure S8.

We further assessed the effect of the degree of dissociation on the low-concentration acetic acid-mediated appressorium formation. Conidia collected from the wild-type strain were suspended in MES buffer adjusted to pH 3.7, 5.0, 7.0, or 8.0. Although the pH of the spore suspension in the absence of any buffer solution was 6.19, all the tested buffer solutions, including that with a pH of 7.0, inhibited appressorium formation (Figure S8A). This result indicated that chemicals in the buffer solution inhibited appressorium formation. However, the rates of inhibition of appressorium formation depended on the pH of the buffer (Figure S8A). Similar results were obtained using phosphate buffer and DMGA buffer, with the greatest decreased in inhibition rate observed at pH 5.0 regardless of the buffer used (Figures S8B and S8C). We next added acetic acid (1 fM) to MES buffer adjusted to pH 4.0, 7.0, or 8.0. Because the pKa value of acetic acid is 4.76, undissociated acid molecules account for approximately 85% of the acid at pH 4.0, 0.5% at pH 7.0, and 0.05% at pH 8.0. In the wild-type strain, appressorium formation was inhibited by buffer solutions containing acetic acid, as shown in Figure S8A. Conversely, in the Δ*cbp1* mutant, the appressorium formation rate was restored only by adding acetic acid to the buffer solution adjusted to pH 4.0 (Figure 3). Therefore, we concluded that acetic acid in an undissociated state acts as a metabolite signal to promote appressorium formation.

### Δ*icl1* Mutation Inhibits Acetic Acid-Mediated Appressorium Formation in the Δ*cbp1* Mutant

**Figure 4:**
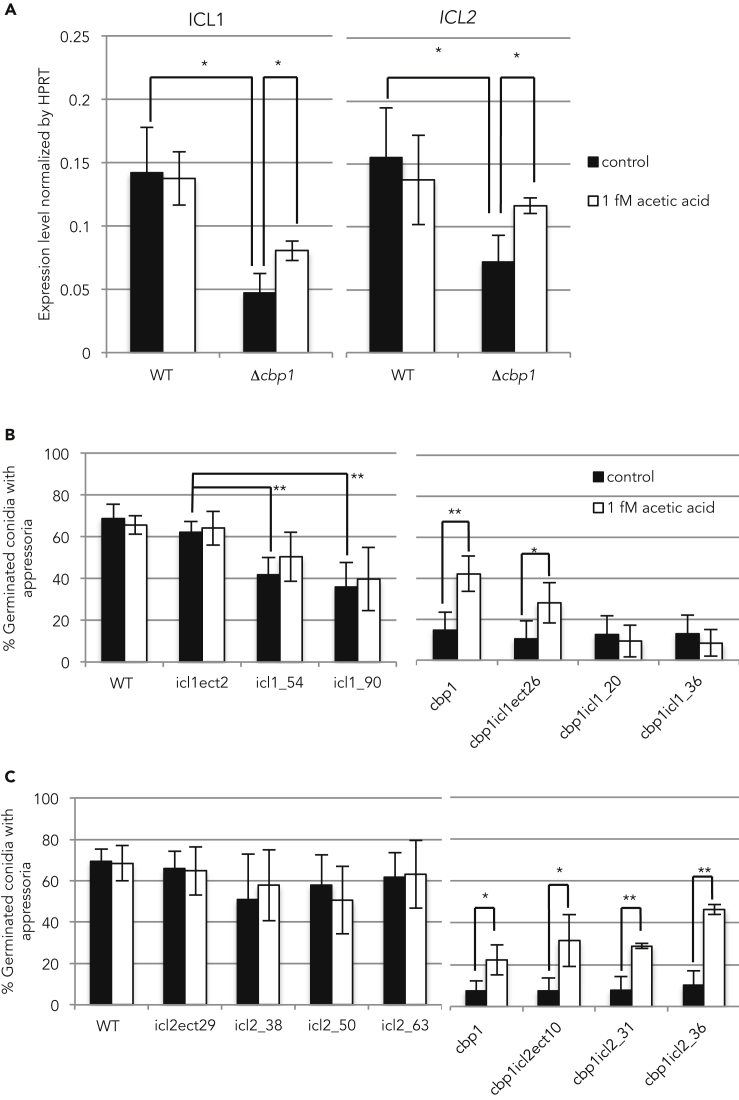
*ICL1* and *ICL2* Are Up-regulated by Adding Acetic Acid and Δ*icl1* Mutation Inhibits Acetic Acid-Mediated Appressorium Formation in the Δ*cbp1* Mutant (A) Transcriptional differences in the *ICL1* (left panel) and *ICL2* (right panel) between the wild-type strain and the Δ*cbp1* mutant. Total RNAs were isolated from the cells at 3 hpi on hydrophobic surfaces. The transcriptional levels of *ICL1* and *ICL2* were normalized to that of *HPRT*. Black bars indicate the expression levels in the presence of H_2_O, and white bars indicate the expression in the presence of 1 fM acetic acid. Error bars indicate standard deviations. Experiments were repeated three times. *p < 0.05 (Student's t test). (B and C) The appressorium formation rate in the *ICL1* (B) and *ICL2* (C) deletion mutants. Black bars indicate appressorium formation in H_2_O, and white bars indicate appressorium formation in 1 fM acetic acid. Left panels show the results in the single mutants, and right panels show the results in the double mutants. Experiments were repeated more than five times. *p < 0.05, **p < 0.01 (Student's t test). Error bars indicate standard deviations. See also Figures S1 and S8.

Cytosolic free fatty acids are converted into acetyl-CoA by the acyl-CoA synthase protein family and are used in various cellular activities and metabolism. Plants, archaea, bacteria, protists, fungi, and nematodes possess a glyoxylate cycle, which is an anaplerotic pathway of the TCA cycle. The glyoxylate cycle allows for the growth on C_2_ compounds as a sole carbon source by bypassing the CO_2_-generating steps of the TCA cycle because the end products of this bypass can be used for gluconeogenesis (Lorenz and Fink, 2001, Dunn et al., 2009, Strijbis and Distel, 2010). Although some enzymes of the glyoxylate cycle overlap with those of the TCA cycle, Icl is a unique enzyme of this bypass pathway. In *M. oryzae*, expression of *ICL1* was elevated during the infection process, and a Δ*icl1* mutant showed a delay in conidial germination, appressorium formation, and cuticle penetration (Wang et al., 2003). Thus, we compared the transcriptional levels of the *ICL1* and its homolog (*ICL2)* between the Δ*cbp1* mutant and the wild-type strain. The expression levels of these genes were significantly reduced in the Δ*cbp1* mutant compared with the wild-type strain (Figure 4A black bars). Meanwhile, the transcriptional reduction of *ICL1* and *ICL2* in the Δ*cbp1* mutant was restored by adding 1 fM acetic acid (Figure 4A, white bars).

We further analyzed the relationship between the glyoxylate cycle and acetic acid using Δ*icl1,* Δ*icl2,* Δ*cbp1*Δ*icl1,* and Δ*cbp1*Δ*icl2* mutants. All single and double mutants grew normally on the medium containing glucose (Figure S9). However, the Δ*icl1* mutants failed to grow on the medium containing sodium acetate as a sole carbon source (Figure S9A). The Δ*icl2* mutants grew on this medium to the same level as the wild-type strain (Figure S9B). These data suggested that Icl1 is vital for the glyoxylate cycle, whereas Icl2 is not involved. The Δ*icl1* mutants also showed a decrease in the appressorium formation rate compared with that in the control strain. In the Δ*icl1* single mutant and Δ*cbp1*Δ*icl1* double mutant, appressorium formation could not be restored by adding acetic acid (Figure 4B). Because these mutants as well as the wild-type strain contain *ICL2*, the data suggested that Icl2 is not involved in acetic acid-mediated appressorium formation. Actually, the Δ*icl2* and Δ*cbp1*Δ*icl2* mutants exhibited the same phenomena as those observed in the ectopic strain and parent strains (Figure 4C). These results suggested the possibility that the low concentration of acetic acid directly or indirectly activates the glyoxylate cycle and induces appressorium differentiation in *M. oryzae*.

## Discussion

In this study, we showed that acidification occurs at the tip of the germ tube during appressorium formation and that an extremely low concentration of acetic acid induces appressorium formation in *M. oryzae*. We concluded that the undissociated state of low-molecular-weight acetic acid triggers a metabolic change and cell differentiation because the low concentration of acetic acid (≤1 μM) did not affect the pH value. Acetic acid has to be in a dissociated state to change the pH, as observed by acidification at the tip of the germ tube. Because the undissociated state of acetic acid could not fully complement the phenotype in the Δ*cbp1* mutant, it seems that Cbp1 can produce a sufficient amount of the dissociated state of acetic acid to change the pH, and the undissociated state of acetic acid also plays an important role in inducing appressorium formation. The expression of *ICL1* and *ICL2* in the Δ*cbp1* mutant was up-regulated by adding acetic acid, whereas the expression of these genes remained unchanged in the wild-type strain. We hypothesized that the expression of *ICL1* and *ICL2* in the wild-type strain was already up-regulated by the CDA activity of Cbp1 before the addition of acetic acid.

Our results are consistent with previous reports that chitosan converted from chitin accumulates at the tips of germ tubes during appressorium formation (Kuroki et al., 2017, Geoghegan and Gurr, 2016). Geoghegan and Gurr reported that chitosan produced by CDA activity is required for surface sensing and germling morphogenesis in *M. oryzae* (Geoghegan and Gurr, 2016). In this report, the chitosan solution was prepared by dissolving in acetic acid, and acetic acid was removed before use by dialysis because chitosan is not dissolved in water. In our opinion, based on the principle of dialysis, it would be expected that the low concentration of acetic acid would remain in the chitosan solution and would affect appressorium formation. Furthermore, chitosan is a macromolecular cell wall component of *M. oryzae* that is additionally covered by glucan, mannan, and hydrophobin; therefore, it also remains unclear how exogenous chitosan permeates through the cell wall and behaves as endogenous chitosan. However, it is experimentally challenging to demonstrate which factors are important for appressorium induction at this stage.

We focused on the glyoxylate cycle and discovered that acetic acid possibly plays a key role in activating this cycle, which results in the induction of appressorium differentiation. From these results, we supposed that the Cbp1-catalyzed conversion of chitin into chitosan is required for the simultaneous release of acetic acid that acts as a mediator and/or signal to specifically trigger the glyoxylate cycle and cell differentiation. In many bacterial and fungal pathogens, the glyoxylate cycle plays an important role in pathogenesis, indicating that these pathogens need to develop in glucose-deficient environments, such as the leaf surface for *M. oryzae* and phagocytic macrophages for the human pathogenic fungus *Candida albicans* (Lorenz and Fink, 2001, Wang et al., 2003, Dunn et al., 2009). The use of acetic acid derived from the degradation of the cell wall as an alternative carbon source is a reasonable strategy for microbial pathogens to evade host defense systems and survive under such conditions. The carbohydrate esterase family 4, which includes chitin and peptidoglycan deacetylases, is conserved among most pathogenic microbes (Aragunde et al., 2018). *Cryptococcus neoformans*, which causes meningoencephalitis, has three CDAs, and these enzymes convert chitin into chitosan, which is involved in cell wall integrity and virulence (Baker et al., 2011). The gram-positive facultative intracellular pathogen, *Listeria monocytogenes*, is able to replicate in macrophages, and cell wall modification via the peptidoglycan *N*-deacetylase is necessary for lysozyme resistance and virulence. The presence of carbohydrate esterase family 4 genes in many pathogenic microbes suggests that the use of acetic acid obtained from the pathogen's own cell wall for the activation of the glyoxylate cycle is perhaps a general mechanism of the infection process. However, in *M. oryzae*, the Δ*cbp1* mutant formed normal appressoria on the plant surface and infected rice cells in the absence of Cbp1 or other CDA activities (Kamakura et al., 2002, Geoghegan and Gurr, 2016). On the artificial hydrophobic plate, Msb2 and Pth11 recognize surface hardness and hydrophobicity, respectively, whereas on the plant surface, plant-specific cues such as plant wax and cutin can also be perceived by *M. oryzae* proteins Msb2, Pth11, and Sho1 (Jiang et al., 2018). After receiving these cues, appressorium formation is induced via pivotal transduction, the cAMP-dependent kinase, and MAP kinase signaling cascades. It is thought that these signal transductions are common on the plant and hydrophobic surfaces. As such, the recognition of plant-specific cues would complement the loss of Cbp1 activity and allow infection of plant cells (Geoghegan and Gurr, 2016). A previous study showed that activation of the cAMP pathway (treatment with IBMX) restored appressorium formation in the Δ*cbp1* mutant (Kamakura et al., 2002). In the Δ*icl1* mutant, appressorium formation capacity on the hydrophobic surface and pathogenicity were dramatically reduced. The low concentrations of acetic acid could also up-regulate *ICL1* in the Δ*cbp1* mutant. In addition, a Δ*msb2*Δ*cbp1* double mutant completely lost the ability to form appressoria and was avirulent, although a Δ*msb2* single mutant retained some pathogenicity (Wang et al., 2015). Together, these findings suggest that the recognition of cues and/or initial signals differs depending on whether *M. oryzae* is on the plant or the hydrophobic surface, and that cell wall-derived acetic acid is related to this process. However, further analysis is required to identify and integrate the complicated signaling pathways influenced by acetic acid in *M. oryzae*.

Gut commensal microbe-derived butyrate is known to induce the cell differentiation of colonic regulatory T cells via histone H3 acetylation (Furusawa et al., 2013). We also found that butyric acid did not significantly improve appressorium formation in the Δ*cbp1* mutant, despite the pKa value being nearly equal to that of acetic acid. Our result suggests that the mechanism of acetic acid-induced cell differentiation differs from that of butyrate, although it is reported that acetate is involved in histone H3 and H4 acetylation in eukaryotes (Gao et al., 2016, Jin et al., 2017). Appressorium formation was restored not only by acetic acid but also by propionic acid and sorbic acid. During the infection process, lipid bodies, which are an abundant form of energy storage in germinating conidia, move to the tip of the germ tube and are rapidly degraded for glycerol biosynthesis and appressorium turgor generation (Wilson and Talbot, 2009). This lipolysis process leads to β-oxidation of fatty acids for the synthesis of acetyl-CoA, which is metabolized in the glyoxylate cycle (Wang et al., 2003, Wang et al., 2007, Bhambra et al., 2006, Ramos-Pamplona and Naqvi, 2006). We therefore proposed that differences in the restoration ability of the appressorium formation rates resulted from the degree of cell permeability, which was dependent on the pKa. The *FAR2* gene, encoding a highly conserved member of the Zn_2_-Cys_6_ family of transcriptional factors, regulates lipid substrate utilization by *M. oryzae*. A *FAR2* deletion mutant could not grow on any short-chain fatty acids including propionate and acetate as the sole carbon source (bin Yusof et al., 2014). Moreover, the deletion of this gene significantly decreased the expression of genes associated with fatty acid β-oxidation (*MFP1*), acetyl-CoA translocation (*PTH2*), peroxisomal biogenesis (*PEX6*), and the glyoxylate cycle (*ICL1*) (bin Yusof et al., 2014). These genes are all involved in pathogenicity and appressorium differentiation in *M. oryzae* (Wang et al., 2003, Wang et al., 2007, Bhambra et al., 2006, Ramos-Pamplona and Naqvi, 2006). Acetate is converted into acetyl-CoA by the catalytic reaction of the acetyl-CoA synthetase (*ACS2* and *ACS3*). The expression of these genes was induced by acetate in the wild-type strain, but not in the Δ*far2* mutant (bin Yusof et al., 2014). These findings indicate that Far2 regulates *ACS2* and *ACS3* in response to short-chain fatty acids, and this was consistent with the fact that fatty acids could also restore appressorium formation in the Δ*cbp1* mutant. The effect of acetic acid, propionic acid, and sorbic acid on redirecting the metabolic flux should be addressed in future analyses.

As described earlier, acetyl-CoA produced from acetic acid is likely to be required by various metabolic mediators to carry out growth and infection functions, such as cell wall, amino acid, and glycerol biosynthesis and appressorium differentiation. However, in this study, the phenotypic restoration and up-regulation of *ICL1* in the Δ*cbp1* mutant was observed by adding an extremely low concentration of acetic acid (1 fM). This level of acetic acid appears to be insufficient for infection-related cell remodeling because acetic acid released at the tip of the germ tube by CDA activity is sufficient to detect a pH shift. This suggests the existence of (a) mediator(s) and/or amplifier(s) to trigger the activation of the glyoxylate cycle and cell differentiation. A recent study revealed that the external application of acetic acid enhanced drought tolerance in *Arabidopsis*, rapeseed, maize, rice, and wheat (Kim et al., 2017). Plants triggered a dynamic metabolic flux conversion from glycolysis into the acetate biosynthetic pathway, comprising pyruvate decarboxylase PDC1 and acetaldehyde dehydrogenase ALDH2B7. Treatment with exogenous acetic acid induced *de novo* jasmonate synthesis and histone H4 acetylation, which elevated drought tolerance. The acetate biosynthetic pathway has also been identified in several fungi (Alvarez et al., 1993, Ingram-Smith et al., 2006, Locklngton et al., 1987, Son et al., 2012, Hu et al., 2008). If the low concentration of acetic acid could induce the glyoxylate cycle, exogenous acetic acid and acetic acid released from the microorganisms' own cell wall may be key regulators initiating the first step in redirecting the metabolic flux, and may in turn increase intracellular acetate by the activation of its biosynthetic pathway.

Based on these findings, we propose that the functions of acetic acid potentially connect fundamental metabolism and cell differentiation. The cellular response triggered by simple and common small molecules occurs at the fM order. This biochemical process has previously been described only for hormone signals in animals. However, we have yet to obtain direct evidence that acetic acid is released by Cbp1 activity and that cell wall-derived acetic acid acts as a signaling molecule for metabolic switching and cell differentiation. To confirm these principles, our future work is aimed at identifying the signaling pathways associated with acetic acid and conducting gene expression profiling to confirm these associations. Nevertheless, the findings presented here illustrate a mode of action for simple compounds at a molecular level and may represent a general phenomenon in cellular biology. We hope that this study will prompt further research across various biological fields.

### Limitations of the Study

In the fM order, acetic acid is only present at a concentration of 100 molecules per fungal spore. Following discussions with experts in the fields of chemistry and biochemistry, it was concluded that direct quantitation or detection of CH_3_COOH or CH_3_COO^˗^ is impossible at this concentration. Furthermore, acetic acid generation at the tips of the germ tubes seemed to occur both spatially and temporally in pulses, and it can be extremely difficult to detect acetic acid directly using presently available experimental technology.

## Methods

All methods can be found in the accompanying Transparent Methods supplemental file.

## Supplemental Information

Document S1. Transparent Methods, Figures S1–S9, and Table S1
