# Supplementary material for: Extremely Low Concentrations of Acetic Acid Stimulate Cell Differentiation in Rice Blast Fungus
Source: iScience. 2019 Dec 19;23(1):100786. doi: 10.1016/j.isci.2019.100786 (PMC6941858; doi:10.1016/j.isci.2019.100786)
Supplement: Document S1. Transparent Methods, Figures S1–S9, and Table S1 [file mmc1.pdf]

**ISCI, Volume *23***

## Supplemental Information

# Extremely Low Concentrations of Acetic Acid Stimulate Cell Differentiation in Rice Blast Fungus

**Misa Kuroki, Yuriko Shiga, Megumi Narukawa-Nara, Takayuki Arazoe, and Takashi Kamakura**

Table S1. pKa of each acid used in this study (Related to Figure 2 and Figure S7).

| | pKa | | |
|---|---|---|---|
| Propionic acid | 4.88 | | |
| Butyric acid | 4.82 | | |
| Acetic acid | 4.76 | | |
| Sorbic acid | 4.76 | | |
| Succinic acid | 4.2 | 5.6 | |
| Lactic acid | 3.86 | | |
| Formic acid | 3.75 | | |
| Citric acid | 3.09 | 4.75 | 6.41 |
| Malonic acid | 2.83 | 5.69 | |
| Glycine | 2.34 | | |
| Phosphoric acid | 2.12 | 7.21 | 12.67 |
| Oxalic acid | 1.27 | 4.27 | |
| Hydrochloric acid | -8 | | |

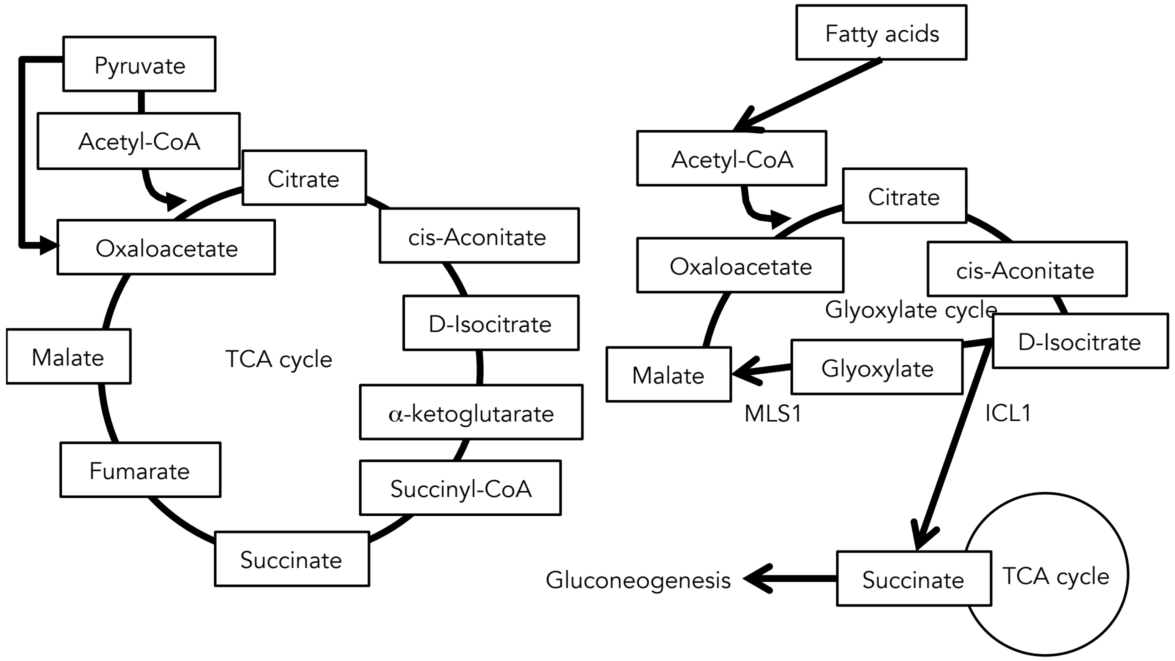


Figure S1 Schematic representation of the TCA cycle and the glyoxylate cycle (Related to Figure 4). Some components of the glyoxylate cycle overlap with those of the TCA cycle. ICL1 and MLS1 are specific to the glyoxylate cycle. Instead of isocitrate dehydrogenase and keto-glutarate dehydrogenase of the TCA cycle, ICL catalyzes the conversion of isocitrate into glyoxylate and succinate and glyoxylate is further catalyzed by MLS, which results in malate. Succinate generated by the glyoxylate cycle is used in gluconeogenesis.

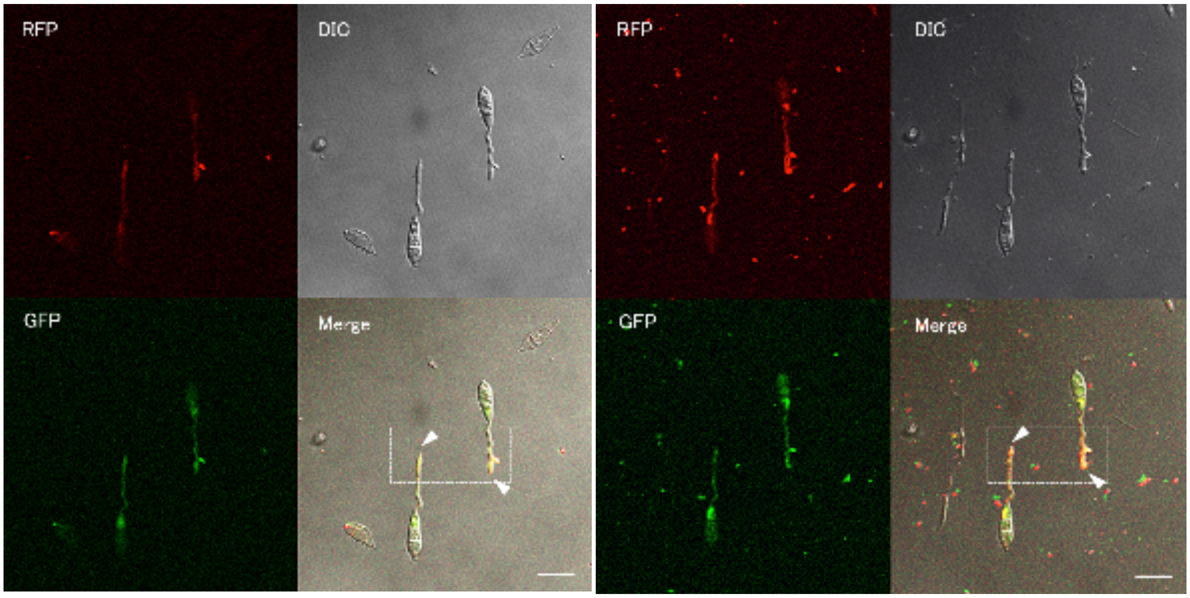


Figure S2 Observations of the pH shift during germ tube elongation in the wild-type strain (Related to Figure 1).

Confocal observations of the pH shift during germ tube elongation in the wild-type strain expressing the pHusion system. Shown are confocal images at 2 hours post-inoculation (hpi) (left panel) and 4 hpi (right panel). Arrowheads point to the tip of the germ tube. Scale bar indicates 20 μm.

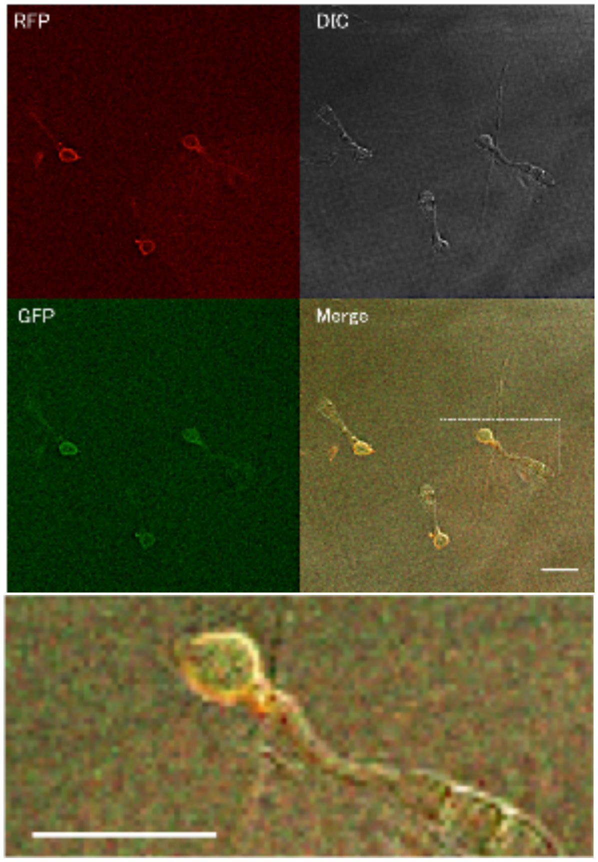


Figure S3 Observations of the pH shift during appressorium formation in the wild-type strain (Related to Figure 1).

Confocal observations of the pH shift during appressorium formation in the wild-type strain expressing the pHusion system. Magnified views of the region are indicated by a white square and are shown below (lower panels). Arrowheads point to the tip of the germ tube. Scale bar indicates 20 μm.

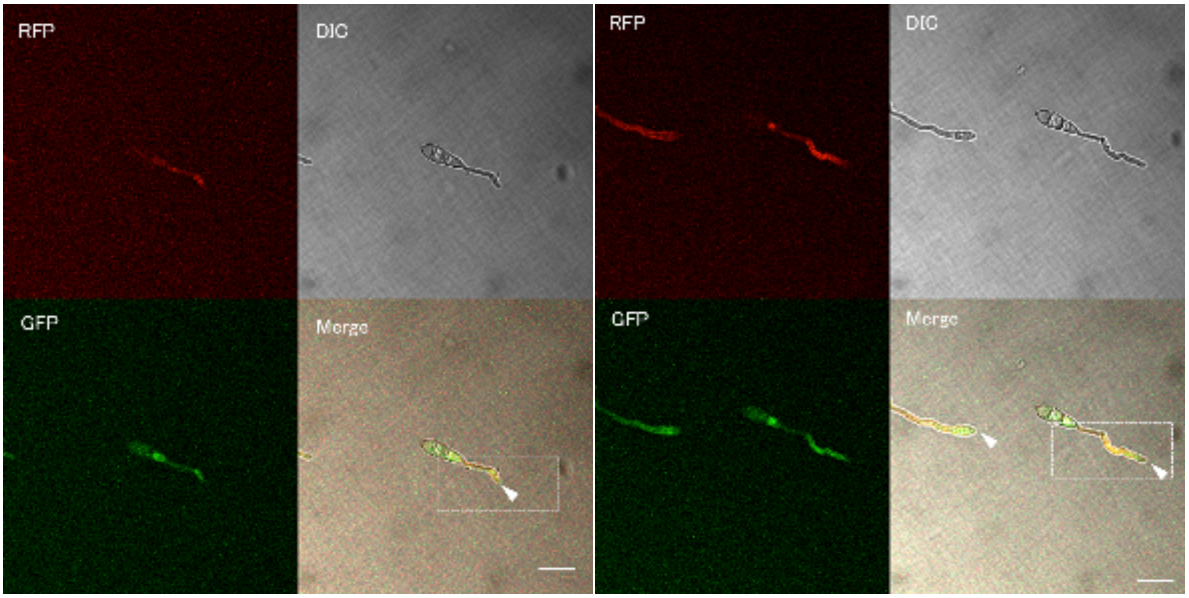


Figure S4 Observations of the pH shift during germ tube elongation in the Δ*cbp1* mutant (Related to Figure 1).

Confocal observations of the pH shift during germ tube elongation in the Δ*cbp1* mutant expressing the pHusion system. Shown are confocal images at 2 hours post inoculation (hpi) (left panel) and 4 hpi (right panel). Arrowheads point to the tip of the germ tube. Scale bar indicates 20 μm.

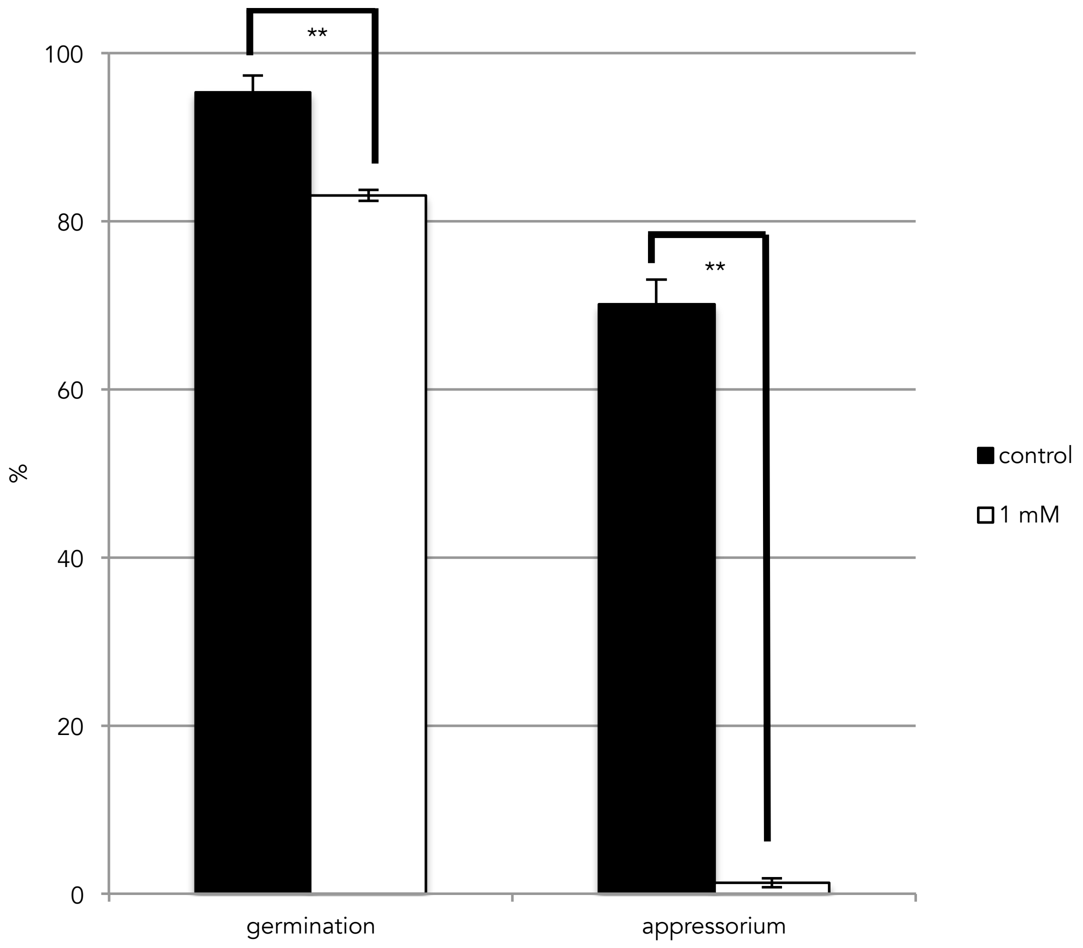


Figure S5 Germination and appressorium formation were inhibited by 1 mM acetic acid (Related to Figure 2).
Bars indicate the germination rate and the appressorium formation rate without acetic acid (black bars) and with 1 mM acetic acid (white bars). Experiments were repeated three times. ** $P < 0.01$ (Student's t-test). Error bars show standard deviations.

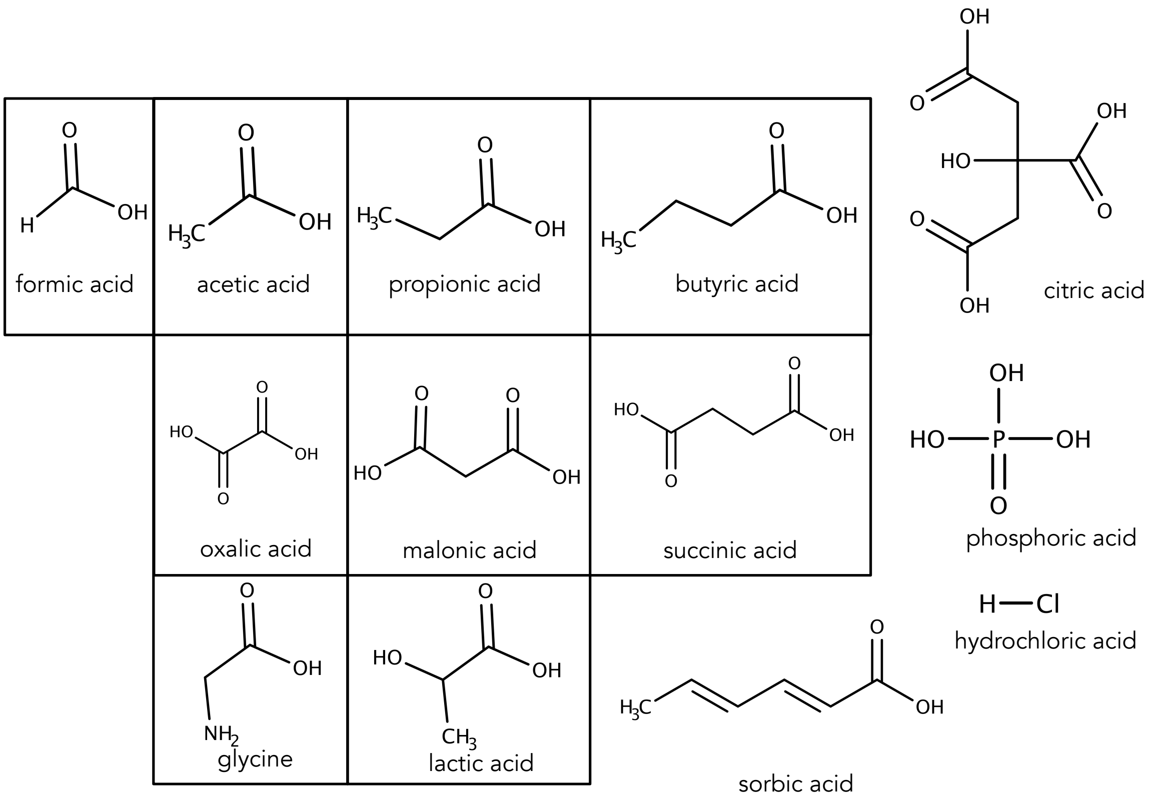


Figure S6 Chemical structure of each acid used in this study (Related to Figure 2 and Figure S7). Formic acid, propionic acid and butyric acid resemble acetic acid in structure. Oxalic acid and glycine are a modified version of acetic acid. Malonic acid, lactic acid and succinic acid were selected as standards. Sorbic acid and citric acid are mild acids that differ in structure from acetic acid. Phosphoric acid and hydrochloric acid are strong acids.

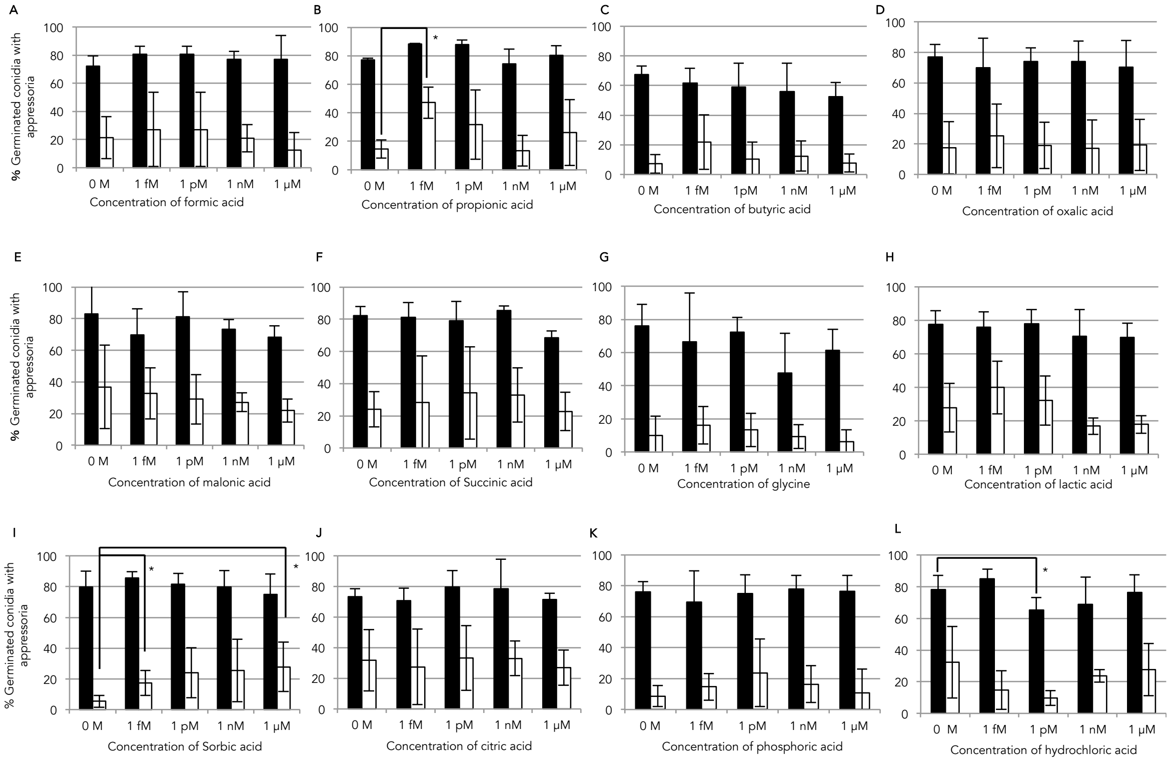


Figure S7 Appressorium formation assay performed in the presence of a range of acids (Related to Figure 2).

Black bars indicate the wild-type strain and white bars indicate the Δ*cbp1* mutant. Some acids were added at each concentration as shown on the x-axis. The figure panels show the amount of appressorium formation in formic acid (A), propionic acid (B), butyric acid (C), oxalic acid (D), malonic acid (E), succinic acid (F), glycine (G), lactic acid (H), sorbic acid (I), citric acid (J), phosphoric acid (K) and hydrochloric acid (L). * $P < 0.05$ compared with 0 M of acid for each strain (Student's t-test). Error bars indicate standard deviations.

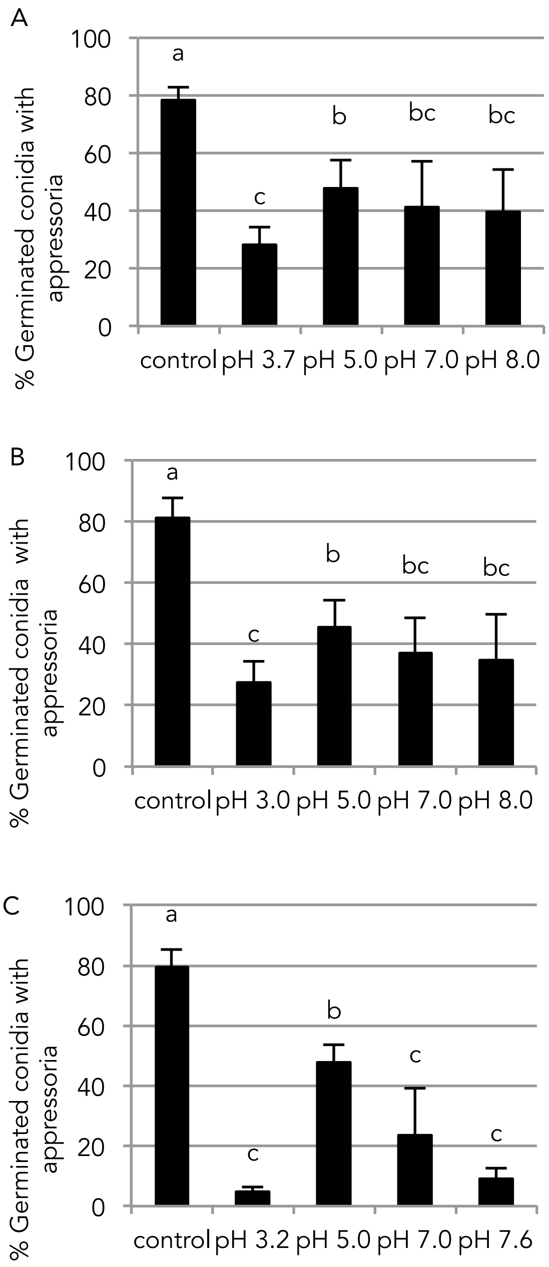


Figure S8. Effects of pH and buffer solutions on appressorium formation (Related to Figure 3).
The pH of MES buffer (A), phosphate buffer (B), and DMGA buffer (C) was adjusted to a range of values, as indicated on the x-axis. Experiments were repeated eight times (A), 10 times (B), or four times (C). Error bars show standard deviations.

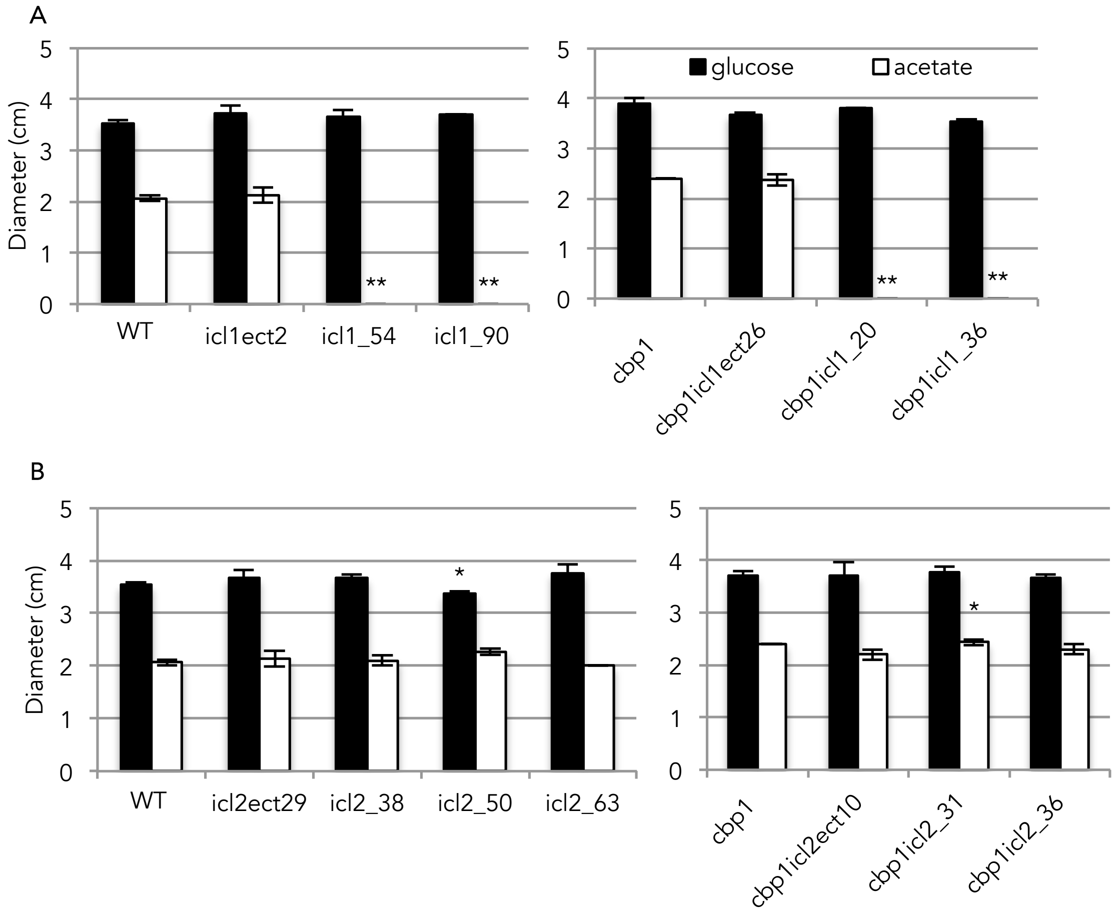


Figure S9 Growth diameter of mutants on medium containing glucose or sodium acetate (Related to Figure 4).

(A) and (B) indicate the growth diameters of ICL1 and ICL2 deletion mutants. Black bars indicate the growth diameter of each strain on medium containing glucose as the sole carbon source and white bars indicate the growth diameter of each strain on medium containing sodium acetate as the sole carbon source. Left panels show single mutants and right panels show double mutants with *CBP1*. Experiments were conducted in triplicate. * $P < 0.05$, ** $P < 0.01$ compared with each ectopic strain (Student's t-test). Error bars indicate standard deviations.

Transparent Methods

Fungal strains and growth media

*Magnaporthe oryzae* wild-type strain P2 was maintained in our laboratory and grown on OM solid medium, containing 5.0% oatmeal (Quaker, Chicago, IL, USA), 0.5% sucrose (Nacalai Tesque, Kyoto, Japan) and 1.5% agar (Nacalai Tesque). The Δcbp1 mutant derived from P2 was generated according to a previously reported protocol (Kamakura et al., 2002). Conidia were obtained by incubation for 2 days under a BLB lamp (Toshiba, Tokyo, Japan) after brushing out aerial mycelia. For the transformation of *M. oryzae*, fungi were incubated in YG liquid medium (0.5% yeast extract (Nacalai Tesque) and 2.0% glucose (Nacalai Tesque)) and the protoplast PEG method was performed as previously reported (Kimura et al., 1995). For measurement of the growth diameter, 50 conidia were dropped into the center of an agar plate of complete media (0.1% (v/v) trace elements, 0.1 mg/L thiamin (Wako Pure Chemical Industries, Ltd., Osaka, Japan), 0.5 µg/L biotin (Nacalai Tesque), 6 g/L $NaNO_3$ (Nacalai Tesque), 0.5 g/L KCl (Nacalai Tesque), 0.5 g/L $MgSO_4$ (Wako Pure Chemical Industries, Ltd.), 1.5 g/L $KH_2PO_4$ (Nacalai Tesque), pH 6.5, adjusted by NaOH, 1.5% agar) containing 6 mM glucose (Nacalai Tesque) or 6 mM sodium acetate (Nacalai Tesque) as the sole carbon source and were incubated at 28°C for 12 days as previously reported (Wang et al., 2003).

Appressorium formation assay

Conidia were suspended in sterilized water using a brush at a concentration of 2.5 conidia/mL. Buffer, acid or water was added to the conidia suspension at the required concentration. In this study, MES buffer, phosphate buffer, DMGA buffer and 14 acids were used. MES buffer

(2-morpholinoethanesulfonic acid monohydrate (Wako Pure Chemical Industries, Ltd.)) was adjusted to pH 3.7, 4.0, 5.0, 7.0 and 8.0. Phosphate buffer ($Na_2HPO_4$ (Nacalai Tesque), $NaH_2PO_4$ (Nacalai Tesque)) was adjusted to pH 3.0, 5.0, 7.0 and 8.0, and DMGA buffer (dimethyl glutarate (Tokyo Chemical Industry Co., Ltd., Tokyo, Japan)) was adjusted to pH 3.2, 5.0, 7.0 and 7.6. Each buffer was added at 10 mM as a final concentration. Acetic acid (Nacalai Tesque), formic acid (Wako Pure Chemical Industries, Ltd.), propionic acid (Wako Pure Chemical Industries, Ltd.), butyric acid (Wako Pure Chemical Industries, Ltd.), oxalic acid (Wako Pure Chemical Industries, Ltd.), glycine (Nacalai Tesque), malonic acid (Nacalai Tesque), lactic acid (Nacalai Tesque), succinic acid (Nacalai Tesque), sorbic acid (Nacalai Tesque), citric acid (Nacalai Tesque), phosphoric acid (Nacalai Tesque) and hydrochloric acid (Nacalai Tesque) were diluted with sterilized water in a step-by-step manner.

Hydrophobic plastic cover slips (Fisher Scientific, Hampton, NH, USA) were wiped with 70% ethanol (Nacalai Tesque) and sterilized water, then placed onto glass slides. Then 20 μL of conidia suspension was placed onto the plastic cover slip and incubated in wet and transparent plastic boxes for 6 h. After incubation, conidia were observed and counted under a light microscope. Appressorium formation rates were calculated as a ratio of germinated conidia with appressoria to germinated conidia without appressoria. Assays were conducted in triplicate and repeated at least three times. Phenotypes were compared according to the average appressorium formation rates and statistical analysis using the Student's t-test.

Generation of pHusion expressing mutants and fluorescent analysis

The eGFP and mRFP fusion protein, pHusion, was cloned from pMP1922-pHusion (Gjetting et al., 2012.), provided from Dr. Alexander Schulz. Then, pHusion was inserted into the pKS01 vector

where it was fused to the signal peptide and S/T cluster derived from Cbp1 cDNA. Then, the CBP1 promoter was inserted at the N-terminus of the signal peptide. The M. oryzae wild-type strain P2 was transformed with this vector and the pCSN43 vector harboring the HPH cassette (Staben et al., 1989). Mutants obtained by this transformation expressed the eGFP–mRFP fusion protein from the CBP1 promoter and localized similarly to Cbp1. The fluorescence of pHusion was observed using the LSM 5 EXCITER (Carl Zeiss AG, Oberkochen, Germany), HFT 488/543/633 beam splitters, an Ar laser (488 nm) and a 505–530 suppression filter for eGFP, and a He-Ne laser (543 nm) and a 560–615 suppression filter for mRFP. The obtained images were analyzed by ImageJ software and fluorescence rates were calculated as the fluorescent intensity of eGFP divided by that of mRFP. Observations were performed at two points, two hours post inoculation (hpi) and 4 hpi. The fluorescence rates at 4 hpi were compared as a ratio of that at 2 hpi. The average rates of fluorescence at 4 hpi compared with 2 hpi were statistically analyzed by the Student's t-test.

## Transcriptional analysis during appressorium formation

Transcription levels were analyzed as previously described (Kuroki et al., 2017). To perform real-time PCR, the following primers were used: ICL1 up (5′ GTG GAA GAT TCT CGA GGG CC 3′) and ICL1 down (5′ GGT ACT TGG CCA TCT GGG TC 3′) for *ICL1*, ICL2 up (5′ CGC CGT ATG CTG ATC TCC TT 3′) and ICL2 down (5′ CCG TGA CCC ATC CAG TTG AA 3′) for *ICL2*.

## Statistical analysis

P values were calculated using Student's t-test as described in the figure legends. P values less than 0.05 were considered to indicate a significant difference.

Supplemental References

Kimura, M., Izawa, K., Yoneyama, K., Arie, T., Kamakura, T., Yamaguchi, I., 1995. A novel transformation system for *Pyricularia oryzae*: adhesion of regenerating fungal protoplasts to collagen-coated dishes. Biosci. Biotechnol. Biochem. 59, 1177–1180.

Staben, C., Jensen, B., Singer, M., Pollock, J., Schechtman, M., 1989. Use of a bacterial hygromycin B resistance gene as a dominant selectable marker in *Neurospora crassa* transformation. Fungal Genet. Rep. 36, 79.
